# Prevalence, incidence and burden of health problems across playing positions in elite male handball players: a 45-week prospective cohort study

**DOI:** 10.1136/bmjsem-2025-002460

**Published:** 2025-04-05

**Authors:** Kristina Drole, Aglaja Busch, Armin Paravlic, Mojca Doupona, Kathrin Steffen

**Affiliations:** 1 Faculty of Sport, Institute of Kinesiology, University of Ljubljana, Ljubljana, Slovenia; 2 School of Health Professions, Division of Physiotherapy, Bern University of Applied Sciences, Bern, Switzerland; 3 Faculty of Sport Sciences, Masaryk University, Brno, Czech Republic; 4 Oslo Sports Trauma Research Center, Norwegian School of Sports Sciences, Oslo, Norway; 5 Norwegian National Unit for sensory loss and mental health, Oslo University Hospital, Oslo, Norway

**Keywords:** Injury, Illness, Handball, Surveillance, Athlete

## Abstract

**Objectives:**

To describe the prevalence, incidence and burden of injuries and illnesses, including their patterns (mechanisms, affected body parts/organ systems) across playing positions (wing, back, line and goalkeeper) in elite adult male handball players.

**Methods:**

The Slovenian version of the Oslo Sports Trauma Research Center Questionnaire on Health Problems (OSTRC-H2-SLO) was used to record health problems (HP) weekly during the 45-week handball season 2022/23.

**Results:**

The study included 189 athletes (age: 23.3±4.4 years). With a weekly response rate of 93%, the mean weekly prevalence of HP was 13.3% (95% CI: 12% to 15%). The overall incidence was 2.2 HP per player per year (95% CI: 1.9 to 2.4), with a cumulative 3318 days lost and a mean time loss of 10.7 days per problem. Acute injuries represented the highest prevalence, incidence and more than 4× greater burden than overuse injuries and illnesses. The knee was the most frequently injured site for both acute and overuse injuries. For acute injuries, the ankle was the second most affected site, while the pelvis/lower back and shoulder were common in overuse injuries. Respiratory illnesses comprised 48% of all illnesses. Wings had the highest prevalence (17%), while backs exhibited the highest incidence (0.99 HP per player per year 95% CI 0.84-1.17), and goalkeepers faced the longest time-loss per HP.

**Conclusion:**

Our findings emphasise the need for position-specific medical care and prevention programmes, targeting knee, ankle, pelvis/lower back, shoulder and respiratory tract. Wings and backs require particular attention due to their high prevalence and burden, while goalkeepers need specialised rehabilitation protocols.

**Trial registration number:**

NCT05471297.

**WHAT IS ALREADY KNOWN ON THIS TOPIC**

**WHAT THIS STUDY ADDS**

- Acute injuries represented the highest prevalence, incidence and more than 4× higher burden than overuse injuries and illnesses.
- The knee emerged as the most commonly injured location, representing more than one-third of acute injuries, which is the highest rate observed in the literature across all sports.
- Wings experience the highest prevalence of health problems, while backs have the highest incidence and burden, and goalkeepers face the longest time-loss per health problem.

**HOW THIS STUDY MIGHT AFFECT RESEARCH, PRACTICE OR POLICY**

- The findings emphasise the need for targeted, position-specific injury prevention and management strategies in handball, with a particular focus on knee, ankle, pelvis/lower back, shoulder and respiratory health.

## Introduction

 Nowadays, handball players confront rising competition demands characterised by higher intensities, increased speed, game dynamics and a greater frequency of actions.^1 2^ The increased physical demands place greater strain on athletes’ bodies throughout the season, which requires them to maintain higher fitness levels.^3^ The intensified training regimen is further compounded by the increasing number of international competitions, necessitating athletes to dedicate more time and effort to maintain their competitive edge. These demands might lead to increased cumulative fatigue and expose athletes to a set of health challenges, including injuries, illnesses and the risk of overtraining syndrome.

While scientific literature has begun to address these health concerns in various sports disciplines,^4^,^6^ the field of handball remains relatively underexplored. There are studies investigating the injury risk during championships^7^,^9^ and Olympic games^10^,^14^ as well as through retrospective^15^ and prospective cohort studies,^16^,^20^ over a one-season course or longer. Although a recent study investigated the health problems of youth handball players,^20^ there is a lack of literature on comprehensive health status examination including both injuries and illnesses in adult handball players. Additionally, the literature lacks data on injury incidence and burden for specific handball positions, a critical factor in understanding the distinct injury characteristics associated with each playing position.^21^

The aim of this study was to describe the prevalence, incidence and burden of injuries and illnesses and their patterns (mechanisms, body parts/organ systems) in elite adult male handball players throughout the 2022/2023 handball season. The secondary aim was to describe the incidence, time loss and burden according to handball playing positions.

## Methods

### Study design and participants

We structured the study as a prospective cohort study and conducted it in accordance with the Strengthening the Reporting of Observational Studies in Epidemiology guidelines^22^ and the Checklist for Statistical Assessment of Medical Papers.^23^ The study was implemented following the principles outlined in the latest version of the Declaration of Helsinki and received formal approval from the National Medical Ethics Committee of Slovenia (approval number: 0120-109/2022/3).

The study population was male handball players participating in the first Slovenian men’s handball league. We determined the sample size prospectively to fulfil the specific requirements of the project.^24^ Recruitment was facilitated through collaboration with the Slovenian Handball Federation and the coaches of various teams. We presented the study objectives and procedures to the federation and team coaches, who subsequently extended invitations for participation to their athletes. Ten out of 12 handball clubs (83%) agreed to participate, yielding a cohort of 189 athletes competing at an elite level. The inclusion criteria for this study required participants to be male handball players actively competing in the first Slovenian handball league, aged 18 years or older. The participants who met the criteria provided written informed consent to participate.

### Definitions

We defined a health problem as any condition that has caused a reduction of the full state of health, regardless of its impact on sports participation or performance and whether the athlete has sought medical help. This can include, but is not limited to, injuries, illnesses, pain or mental health conditions.^25^ We categorised health problems as either injuries or illnesses and defined them according to common consensus on research methodology.^26^ Handball-specific training included all sessions focusing on handball technique and tactics, while strength and conditioning training referred to sessions which included resistance and/or endurance training. Matches were defined as organised games against other teams, encompassing official and friendly matches.

### Materials and procedure

We monitored the athletes for a period of 45 weeks, from 19 July 2022, to 2 June 2023, covering the entire 2022/2023 handball season. We used the Slovenian version of the Oslo Sports Trauma Research Center Questionnaire on Health Problems (OSTRC-H2-SLO)^27^ to capture health problems on a weekly level. The questionnaire and its scoring methodology are described in detail previously.^25 27^ Health problems were categorised as substantial if they resulted in moderate to severe reductions in training volume or performance or if an athlete could not participate in the training process due to a health problem.^28^ Time loss health problems were those that caused the player to miss training sessions or matches. If multiple health issues occurred within the same week, each problem was evaluated individually, starting with the most severe.

Participants completed the OSTRC-H2-SLO questionnaire weekly through an online platform 1ka (www.1ka.si). A reminder with a link to the online questionnaire was sent every Sunday, and if not completed, a follow-up reminder was sent within 2 days. Athletes reporting a health problem were contacted by a medical professional to verify the reported health problem. In addition to the OSTRC-H2 questionnaire, athletes, in collaboration with their support staff, provided weekly reports on their load (in hours) across four categories:

1. Training load: Sport-specific (handball) training and strength and conditioning sessions.
2. Competition load: The duration of matches played was recorded in minutes and converted to hours.
3. Academic load: Time spent on lectures, exams, practical courses, and studying.
4. Workload: Time dedicated to any additional employment alongside their athletic career.

### Statistical analyses

We performed all statistical analyses using R statistical software (V.3.6.1). The response rate was determined by dividing the number of completed questionnaires by the total number distributed. The weekly prevalence of health issues was calculated by dividing the number of players reporting at least one health problem by the number of questionnaire responses. The overall incidence of health problems was expressed as the number of problems per athlete per year, while for injury cases, we additionally calculated the number of injuries per 1000 hours of exposure.^29^ Exposure was defined as time spent in handball training, strength and conditioning training and matches. The average weekly severity score was presented as averaging the score of all athletes who reported a problem. The health problem burden was calculated as the number of time loss days per athlete per year. For all the above calculations, recurrent problems were counted as the same event if they were deemed by the medical staff to be exacerbations of an unresolved injury/illness or a chronic injury/illness.

We performed the sub-analyses to assess health problem patterns according to playing positions, defined as back, wing, line and goalkeeper. For each group, we calculated the injury incidence, time loss and injury burden.

### Equity, diversity and inclusion statement

The authors are based in Slovenia, Switzerland and Norway; consist of both women and men; and include both junior and senior researchers. The authors have backgrounds as physiotherapists and kinesiologists. We acknowledge that while our study was conducted on first men’s handball league players, it excludes women and athletes playing different sports.

### Patient and public involvement

The study participants were not involved in the design, conduct, reporting or dissemination of this research.

## Results

### Participants and response rate

The final cohort was comprised of 189 players from 10 handball clubs: age, 23.3±4.4 years (range 18–41 years); height, 188.9±6.3 cm; weight, 92.4±11.3 kg (body mass index 25.9±2.3 kg/m²); years of handball experience, 13.8±4.4 years; weekly training load, 8.6±4.4 hours; competition load, 0.3±0.4 hours; academic load, 3.7±7.6 hours; and work load, 5.2±12.3 hours. The players responded to 6980 out of 7505 questionnaires distributed, which yielded a response rate of 93%. A total of 33 athletes dropped out due to various reasons, including transfers (n=6), loans (n=5), transitioning to a goalkeeper coach (n=1) and change of the main coach (n=21). However, they were included in the analysis up until their dropout.

### Prevalence

Four out of five players (79%) reported at least one health problem over the course of the season. In total, 316 new health problems were reported, of which 53%, 28% and 20% were acute injuries, overuse injuries and illnesses, respectively.

The average weekly prevalence, incidence, time loss and burden for all health problems, substantial health problems and time loss health problems with 95% CIs for specific playing positions and altogether are summarised in table 1.

**Table 1:** Prevalence, incidence, time loss and burden of health problems for overall and specific playing positions

Category	Position	Prevalence (95% CI)	Incidence per athlete per year (95% CI)	Incidence per 1000 hours (95% CI)	Mean time loss (95% CI)	Burden (95% CI)
All health problems(n=316)	Overall (n=189)	13% (12 to 15%)	2.19 (1.96 to 2.45)	4.42 (3.95 to 4.93)	10.50 (7.9 to 13.5)	22.96 (22.19 to 23.75)
	Back (n=82)	12% (9 to 14%)	0.99 (0.84 to 1.17)	2.0 (1.69 to 2.35)	8.20 (6.09-10-80)	8.14 (7.68 to 8.61)
	Wing (n=48)	17% (15 to 19%)	0.53 (0.43 to 0.66)	1.08 (0.86 to 1.34)	12.70 (6.77 to 20.50)	6.78 (6.36 to 7.21)
	Line (n=29)	14% (12 to 16%)	0.40 (0.31 to 0.52)	0.8 (0.61 to 1.03)	9.93 (4.33 to 17.90)	4.01 (3.69 to 4.34)
	Goalkeeper (n=30)	12% (10 to 13%)	0.27 (0.2 to 0.37)	0.55 (0.39 to 0.74)	15.20 (5.77 to 28.40)	4.12 (3.80 to 4.46)
Substantial		9% (8 to 10%)				
Time loss		12% (10 to 13%)				
Acute injuries (n=166)	Overall	9% (8 to 10%)	1.15 (0.99 to 1.34)	2.32 (1.99 to 2.70)	13.70 (9.75 to 18.50)	15.73 (15.10 to 16.39)
	Back		0.58 (0.47 to 0.72)	1.18 (0.94 to 1.45)	10.90 (7.49 to 15.00)	6.35 (5.94 to 6.77)
	Wing		0.21 (0.14 to 0.29)	0.42 (0.29 to 0.59)	24.30 (10.20 to 43.30)	5.07 (4.71 to 5.45)
	Line		0.24 (0.17 to 0.33)	0.49 (0.35 to 0.67)	8.57 (3.71 to 16.30)	2.08 (1.86 to 2.33)
	Goalkeeper		0.12 (0.07 to 0.18)	0.24 (0.14 to 0.37)	18.90 (7.82 to 34.40)	2.24 (2.0 to 2.49)
Substantial		6% (6 to 7%)				
Time loss		8% (6 to 9%)				
Overuse injuries (n=88)	Overall	3% (2 to 4%)	0.62 (0.50 to 0.76)	1.23 (0.99 to 1.51)	6.22 (3.35 to 10.9)	3.79 (3.49 to 4.13)
	Back		0.24 (0.17 to 0.33)	0.49 (0.35 to 0.67)	4.26 (2.54 to 6.40)	1.03 (0.88 to 1.21)
	Wing		0.20 (0.14 to 0.29)	0.41 (0.28 to 0.57)	5.86 (3.17 to 9.03)	1.18 (1.01 to 1.37)
	Line		0.09 (0.05 to 0.15)	0.18 (0.10 to 0.30)	15.00 (1.54 to 41.20)	1.35 (1.17 to 1.55)
	Goalkeeper		0.08 (0.04 to 0.13)	0.15 (0.08 to 0.27)	3 (1.64 to 4.91)	0.23 (0.16 to 0.32)
Substantial		1% (1 to 1%)				
Time loss		3% (2 to 4%)				
Illnesses (n=62)	Overall	1% (1 to 2%)	0.43 (0.33 to 0.55)		7.97 (4.06 to 15.0)	3.43 (3.14 to 3.74)
	Back		0.17 (0.11 to 0.24)		4.54 (3.71 to 5.33)	0.76 (0.62 to 0.91)
	Wing		0.12 (0.08 to 0.19)		4.22 (3.56 to 4.89)	0.53 (0.42 to 0.66)
	Line		0.06 (0.03 to 0.11)		7.89 (2.89 to 14.30)	0.49 (0.39 to 0.62)
	Goalkeeper		0.08 (0.04 to 0.13)		21.60 (2.73 to 58.60)	1.65 (1.45 to 1.87)
Substantial		1% (1 to 1%)				
Time loss		1% (1 to 2%)				

### Incidence and burden

The athletes reported a total of 50 778 hours of handball exposure (3675 match and 47 103 handball training hours). Additionally, there were 20 674 hours of strength and conditioning training reported. Overall, athletes experienced 316 health problems which resulted in a cumulative 3318 days lost, with a mean time loss of 10.7 days per problem. The overall incidence was 2.19 (95% CI 1.96 to 2.45) health problems per player per year and 4.42 (3.95–4.93) injuries per 1000 hours of exposure.

**Figure 1:**
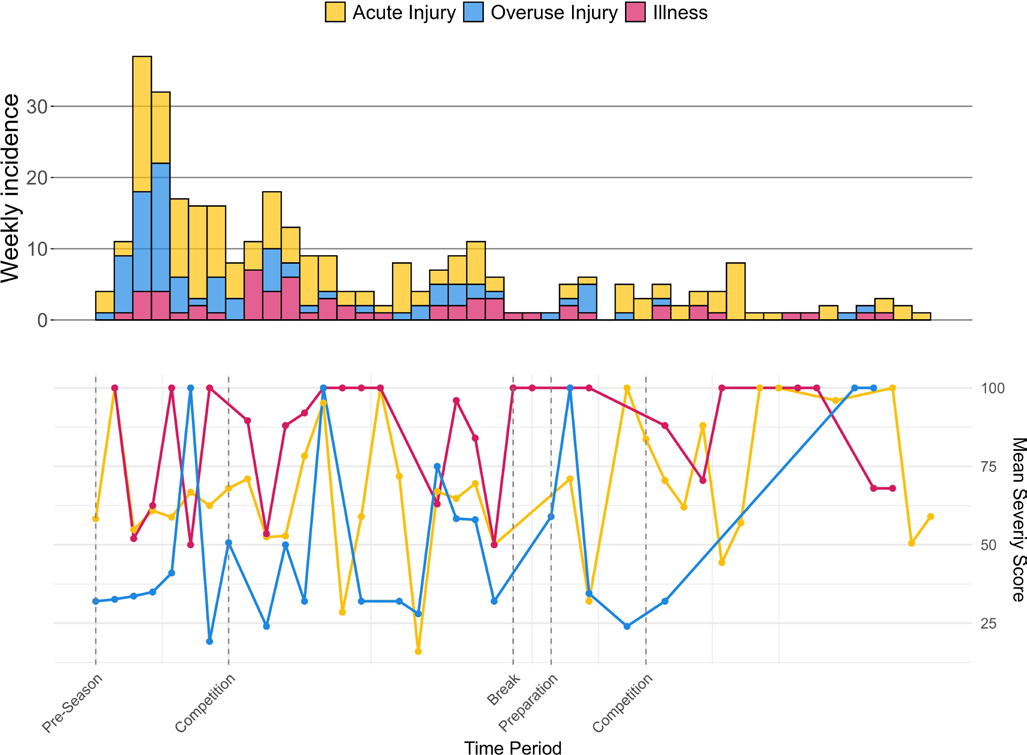
Incidence and severity during the 45 weeks by type of health problem.

The incidence of health problems, including acute injuries, overuse injuries and illnesses, varies across playing positions (table 1) and season period (figure 1).

### Common injury sites and affected organ systems by illness

For both acute and overuse injuries, the knee emerged as the most commonly affected site. Additionally, the ankle was the second most frequent injury site in acute cases, while the pelvis/lower back and shoulder were most frequently injured in overuse injuries (table 2).

**Table 2:** Common injury sites and affected organ systems by illness across playing positions

Body part/organ system	Type	Number (%)	Back	Wing	Line	Goalkeeper
Injury						
Head	Acute	6 (4%)	1 (1%)	1 (3%)	4 (11%)	0
	Overuse	0 (0%)	0	0	0	0
Neck	Acute	4 (2%)	2 (2%)	2 (7%)	0	0
	Overuse	1 (1%)	0	0	1 (8%)	0
Shoulder	Acute	12 (7%)	8 (10%)	2 (7%)	1 (3%)	1 (6%)
	Overuse	12 (14%)	9 (26%)	0	0	0
Upper arm	Acute	1 (1%)	1 (1%)	0	0	0
	Overuse	0	0	0	0	0
Elbow	Acute	2 (1%)	2 (2%)	0	0	0
	Overuse	1 (1%)	0	0	0	1 (9%)
Lower arm	Acute	1 (1%)	1 (1%)	0	0	0
	Overuse	0	0	0	0	0
Wrist	Acute	4 (2%)	2 (2%)	0	1 (3%)	1 (6%)
	Overuse	0	0	0	0	0
Palm/fingers	Acute	9 (5%)	5 (6%)	0	2 (6%)	3 (18%)
	Overuse	0	0	0	0	0
Chest	Acute	7 (4%)	3 (4%)	3 (10%)	1 (3%)	0
	Overuse	0	0	0	0	0
Stomach	Acute	3 (2%)	2 (2%)	1 (3%)	0	0
	Overuse	1 (1%)	0	0	0	0
Pelvis/lower back	Acute	6 (4%)	2 (2%)	0	2 (6%)	2 (12%)
	Overuse	17 (19%)	7 (20%)	0	4 (31%)	1 (9%)
Hip/groin	Acute	6 (4%)	4 (5%)	1 (3%)	1 (3%)	0
	Overuse	8 (9%)	4 (11%)	3 (10%)	0	1 (9%)
Thigh	Acute	21 (13%)	10 (12%)	5 (17%)	4 (11%)	2 (12%)
	Overuse	7 (8%)	1 (3%)	0	1 (8%)	3 (27%)
Knee	Acute	27 (16%)	16 (19%)	5 (17%)	5 (14%)	1 (6%)
	Overuse	21 (24%)	8 (23%)	7 (24%)	3 (23%)	3 (27%)
Lower leg	Acute	6 (4%)	2 (2%)	1 (3%)	2 (6%)	1 (6%)
	Overuse	11 (13%)	2 (6%)	5 (17%)	3 (23%)	1 (9%)
Ankle	Acute	36 (22%)	18 (21%)	4 (13%)	11 (31%)	3 (18%)
	Overuse	3 (3%)	0	1 (3%)	1 (8%)	1 (9%)
Foot	Acute	8 (5%)	2 (2%)	3 (10%)	1 (3%)	2 (12%)
	Overuse	2 (2%)	1 (3%)	1 (3%)	0	0
Other	Acute	6 (4%)	3 (4%)	2 (7%)	0	1 (6%)
	Overuse	4 (5%)	3 (9%)	1 (3%)	0	0
Illness						
Otological	Illness	1 (2%)	0	0	0	1 (9%)
Dermatological	Illness	2 (3%)	0	0	0	2 (18%)
Respiratory	Illness	30 (48%)	17 (71%)	6 (33%)	4 (44%)	3 (27%)
Cardiovascular	Illness	1 (2%)	0	1 (6%)	0	0
Genitourinary	Illness	1 (2%)	0	0	0	1 (9%)
Gastrointestinal	Illness	14 (23%)	5 (21%)	5 (28%)	2 (22%)	2 (18%)
Multiple systems or not otherwise specified	Illness	10 (16%)	1 (4%)	4 (22%)	3 (33%)	2 (18%)
Overall	Illness	3 (5%)	1 (4%)	2 (11%)	0	0

Back players had the highest occurrence of shoulder injuries, with 26% being overuse injuries and 10% acute injuries. Knee injuries followed closely, comprising 23% overuse and 19% acute injuries, while ankle injuries were predominantly acute (21%). Wing players experienced a significant proportion of knee injuries, with 24% being overuse injuries and 17% acute injuries. This was followed by overuse injuries to the lower leg (17%) and acute ankle injuries (13%). For line players, the most common injury was acute ankle injury (31%), followed by overuse knee injuries (23%) and overuse lower leg injuries (23%). Goalkeepers had the highest proportion of overuse thigh (27%) and knee (27%) injuries, with acute ankle injuries (18%) also being prevalent.

Approximately 50% of the reported illnesses were related to the respiratory system, followed by gastrointestinal illnesses (23%), illnesses affecting multiple systems (16%) and overall illness (5%) (table 2).

### Injury mechanisms and activities associated with injury occurrence

For acute injuries, non-contact mechanisms accounted for 46.1% of cases, followed by direct contact injuries at 40%, indirect contact at 11.5% and unspecified mechanisms at 2.4%. The most common causes of acute injuries were collisions with another player (33.9%) and landing incidents (22.4%). Other notable causes included running (12.7%), collisions with other objects (6.7%), take-off events (5.5%), jumping (2.4%), falls (0.6%), and various non-specified causes (15.7%).

## Discussion

This study is the first to comprehensively document health problems, including illnesses, among adult male elite handball players over an entire handball season period of 45 weeks while also examining injury incidence, time loss and burden specific to each playing position. Our findings reveal that, at any given time, approximately 13% of participating players reported a health issue. Acute injuries represented the highest prevalence and more than 4× higher burden than overuse injuries and illnesses. The knee was identified as the most frequently injured site for both acute and overuse injuries. Non-contact mechanisms were responsible for 46.1% of the acute injuries, with collisions and landings being the most common causes. Respiratory system illnesses were the most frequent, followed by gastrointestinal issues.

### Prevalence

The health problem prevalence rate of 79% among Slovenian handball players aligns closely with the 77.9% reported for German handball players,^15^ but it is notably higher than the 57% prevalence found in Icelandic handball players.^19^

The weekly prevalence of health problems in our senior handball players was 13%, which was lower than previously documented with the same methodology in other sports and ages.^45 30^,^33^ Bjorndal and colleagues^20^ reported 53% prevalence in youth handball athletes; however, a higher prevalence of injuries in youth athletes is well-documented in the literature.^31^ Other studies reported a prevalence of 36% in adult Olympic athletes from various sports,^32^ 43% in youth athletes from various sports,^31^ 40% in adult male ice hockey players and 21% in 11–14-year-old tennis players.^33^ However, similar to our results, lower prevalence rates in senior athletes, particularly those competing at the international level, have been observed.^30^ The differences in the prevalence rates could be due to the fact that all teams in the first handball league are mandatory to have a proper support team with a physiotherapist, who handles the athletes’ health problems. Consequently, senior athletes playing at the highest level have better support than youth athletes, with the exception of athletes who are selected to compete for the national teams. Moreover, the majority of Slovenian handball players are not purely professional handball players, but are also having dual or triple careers (simultaneously participating in both sport and education and/or work).

Despite the difference in prevalence rates, both Bjorndal and colleagues^20^ and our study found that injuries were more common than illnesses. However, youth athletes had a higher incidence of overuse injuries,^20^ while our senior athletes experienced more acute injuries. This pattern suggests a potential shift in injury types with age and experience, possibly due to differences in training intensity, physical maturity and injury prevention strategies.

The findings from this study highlight differences in injury incidence, prevalence and the associated time loss and burden across different playing positions, indicating the need for position-specific strategies in injury prevention and management. The highest prevalence of health problems was observed among wings (17%), followed by line players (14%), backs (12%) and goalkeepers (12%), suggesting that wings may have a higher proportion of players experiencing health problems at any given time.

### Incidence and burden

The incidence rate per 1000 hours of exposure in our study was 4.42, which is comparable to the incidence rates in the German (4.3),^15^ but not Icelandic (1.1) league players,^19^ which only accounted for time loss acute injuries. This discrepancy may be attributed to the more comprehensive inclusion of health problems in our study, encompassing both injuries and illnesses. Moreover, Peterson and colleagues documented an incidence of 7.3 injuries per 1000 hours of exposure in football, with substantial variation based on age and skill level.^6^ Additionally, both the study by Peterson and colleagues and our study confirm that a significant proportion of injuries result from non-contact mechanisms. This is promising, as it suggests that many of these injuries may be preventable through targeted prevention programmes in both sports.

In contrast to prevalence, backs exhibited the highest incidence of all health problems, including acute injuries, overuse injuries and illnesses. This indicates that backs are more likely to experience new health issues during the study period,^21^ which could be attributed to the extensive time spent on the court, combined with the demands of intense running, agility and physical contact inherent to their position.^34 35^ Interestingly, despite having the lowest incidence of health problems, goalkeepers experienced the highest time loss from training and competition, with an average of 15.20 days missed per health problem. This suggests that while goalkeepers might incur fewer new injuries, the severity or recovery time of their injuries may be greater. Furthermore, backs with 8.14 days lost per athlete per year demonstrated 1.2× higher burden than wings and 2× higher burden than line players and goalkeepers. This indicates that the cumulative impact of their injuries was more substantial compared with players in other positions. This could be attributed to the unique physical demands placed on back players who spend more time standing still and walking while also performing high-intensity actions,^34^ leading to a greater physiological strain during gameplay.

In contrast, wing players demonstrated a lower incidence of injuries but suffered from more severe consequences when injuries occurred. Longer time loss observed in wing players may be attributed to the dynamic and high-intensity movements required in this position, such as rapid changes of direction and speed. Line players, contributing to both offensive and defensive play, showed a moderate incidence of injuries with a corresponding injury burden. Goalkeepers, on the other hand, had the lowest incidence of injuries but the longest time loss. This unique injury profile for goalkeepers might be explained by the specialised and often high-risk movements they perform, such as diving or rapid lateral shifts, which, although infrequent, can lead to serious injuries requiring extensive rehabilitation. The variation in injury patterns across these positions underscores the importance of tailored conditioning and rehabilitation programmes.

A total of 12% of health problems were time loss, among which 10% were injuries. The severity scores were highest in week 23, which is just at the end of the first competition period (before Christmas holidays). This could be due to several reasons, such as accumulation of load throughout the first part of the season, but also the winter time itself.^36^ During the winter months, athletes seem to be more susceptible to illness, as respiratory infections are on the rise.^37^

### Common injury sites and affected organ systems by illness

The most common injury sites were knee and ankle for acute injuries and knee and shoulder for overuse injuries, which is in agreement with other studies.^6 15 18 19 38 39^ Handball is known for a high incidence of both acute and overuse injuries.^8 40^ Namely, acute injuries predominantly occur in team sports characterised by significant player physical contact with an opponent and in sports with high speeds and elevated risks of falls.^41^ On the contrary, overuse injuries are frequently encountered in endurance sports that demand extensive training periods, as well as in technical disciplines where repetitive motion is common.

Acute injuries in our study were most frequently non-contact, often occurring during landing, while collisions were a frequent cause of acute injuries as well. This finding is consistent with previous research that similarly identified player collisions and landing incidents as common causes of acute injuries.^19^ More than one-third (39%) of acute injuries were located in the knee, which is the highest rate observed in the literature across all sports. Rafnsson and colleagues^19^ and Olsen and colleagues^38^ found one-fourth of injuries were located in the knee, while a lower rate was observed in Brazilian (8.8%) handball players.^18^ However, our results support the fact that approximately 50% of acute injuries contained during handball are located in the knee and ankle.^38^ These variations might be influenced by differences in study populations, injury definitions and data collection methods.

Illnesses, while less frequent than injuries, had a notable impact. An acute illness represents a significant health burden for athletes,^42^ as it can lead to decreased performance, interruption of the training process and even missing out on an important competition. The organ systems affected by acute illness in athletes show a very consistent pattern. Most studies indicate that about 50% of all acute illnesses in athletes during competitions affect the respiratory system, while other affected organ systems include the digestive system,^12 43 44^ skin and subcutaneous tissue, and the genitourinary system.^44^ Our results demonstrate that approximately one-half of all reported illnesses were related to the respiratory system, followed by gastrointestinal issues and multisystem illnesses. This distribution highlights the susceptibility of athletes to respiratory infections, likely due to the high physical demands and frequent travel associated with competitive sports. In team handball, the risk of respiratory tract infections is likely higher due to close player contact and the indoor setting, which further facilitates the virus spread. Research reports that illnesses (most commonly the respiratory tract) account for 33% of lost training sessions. Additionally, acute infections can increase the risk of serious health complications and even sudden death during strenuous exercise;^45^ therefore, illnesses in athletes should not be neglected. It is necessary to pay more attention to monitoring illnesses, not only injuries, and ensure a proper return to sport after illness.

### Clinical implications

This study highlights the need for position-specific injury prevention strategies in elite male handball players. Integrating these findings into athlete care practices can lead to more effective prevention, reduced time loss and improved overall performance. By understanding the position-specific demands and associated health risks, coaches, sports scientists and clinicians can implement interventions that prioritise athlete safety and optimise both individual and team outcomes. Given that non-contact injuries accounted for 46.1% of all acute injuries, implementing targeted prevention strategies could help reduce their occurrence, particularly during the preseason and early-season phases, when injury rates were highest. Structured neuromuscular training, strength and conditioning programmes, and workload management may enhance players’ preparedness and reduce injury risk. Additionally, gradual exposure to high-intensity movements and sport-specific drills could better prepare athletes for in-season demands, minimising the likelihood of injuries early in the season.

### Strengths and limitations

The study shows its strength in the sample, representing the first Slovenian handball league, and the high response rate throughout one season. Additionally, this is the first study to investigate the health problems holistically (including both injuries and illnesses) in adult handball players and presenting injury incidence and burden data according to the playing positions.

There are several limitations to our study. The reliance on self-reporting introduces potential biases and inaccuracies due to the subjectivity of the questionnaires. Additionally, the study only included senior elite male handball players, limiting our ability to compare findings across sexes, ages and levels of play. Comparing our findings with previous studies reveals the complexity of sports injury research. Variations in health problem definitions, data collection methods and study designs make direct comparisons challenging. Moreover, some studies did not have available training exposure^15^ and are therefore not eligible for comparison.

This highlights the need for sport-specific investigations conducted with consistent and comparative methodologies. Future research should delve deeper into the biomechanical and physical demands of each playing position to further refine injury prevention strategies and better understand the causes of positional differences in injury patterns.

## Conclusion

In conclusion, our findings highlight the high prevalence, incidence and burden of acute injuries in adult male handball players, emphasising the need for position-specific medical care and injury and illness prevention programmes, focusing on the knee, ankle, pelvis/lower back, shoulder and respiratory tract. Acute injuries, particularly non-contact injuries of the knee, were the most prevalent and carried the highest burden across all playing positions, necessitating focused attention from the medical team. Wings exhibited the highest prevalence of health problems, while backs had the highest incidence and injury burden, highlighting their susceptibility to new health problems. Goalkeepers, despite having the lowest incidence, faced the longest time-loss per injury, suggesting a need for specialised rehabilitation protocols. Additionally, respiratory illnesses were the most frequent type of illness, underscoring the importance of monitoring and addressing infections, particularly during high-risk periods like winter.

## Data Availability

Data are available upon reasonable request. All data relevant to the study are included in the article or uploaded as supplementary information.
